# Aerobic Exercise Alleviates Oxidative Stress and Inflammation to Attenuate High-Fat Diet-Induced Non-Alcoholic Fatty Liver Disease in *ApoE^-/-^* Mice

**DOI:** 10.3390/metabo16040285

**Published:** 2026-04-21

**Authors:** Liang Zhang, Wenxin Wang, Fengting Zheng, Jialu Weng, Yao Lu, Qingbo Li, Ting Li, Wei Li, Lifeng Wang

**Affiliations:** College of Physical Education and Health Sciences, Zhejiang Normal University, Jinhua 321004, China; zhangliang0433@163.com (L.Z.); 15068671301@163.com (W.W.); 15157060709@163.com (F.Z.); 18994600327@163.com (J.W.); gbiyyl@163.com (Y.L.); lqb1971701428@163.com (Q.L.); tingli@zjnu.edu.cn (T.L.)

**Keywords:** non-alcoholic fatty liver disease, exercise, sestrin2, oxidative stress, inflammation

## Abstract

**Background/Objectives**: The development of non-alcoholic fatty liver disease (NAFLD) is closely linked to oxidative stress and inflammation. Aerobic exercise has been shown to improve NAFLD, although its underlying mechanisms remain incompletely understood. This study utilized *ApoE^-/-^* mice to investigate the role of Sestrin2 in aerobic exercise-induced amelioration of NAFLD. **Methods**: Random assignment of C57BL/6J and *ApoE*^-/-^ mice yielded four groups: C (control), CE (aerobic exercise), AS (*ApoE^-/-^* control), and AE (*ApoE^-/-^* aerobic exercise). Aerobic exercise lasting 12 weeks was administered to the CE and AE groups. Serum biomarkers were analyzed by ELISA, liver tissue morphology was assessed via HE and ORO staining, and macrophage polarization was evaluated through immunofluorescence. Additionally, mRNA and protein expression levels were measured by qPCR and Western blot. **Results**: Aerobic exercise reduced liver wet weight, lipid accumulation, and steatosis in *ApoE^-/-^* mice. Aerobic exercise attenuates hepatic oxidative stress, and upregulated the expression of regulation oxidative stress related gene and proteins of Nrf2, HO-1, CAT, and SOD1 in *ApoE^-/-^* mice. Aerobic exercise promoted a shift in macrophage polarization from the pro-inflammatory M1 phenotype toward the anti-inflammatory M2 phenotype in the liver, and significantly reduced TNF-α and IL-1β levels, accompanied by upregulation of Sestrin2 expression, enhanced AMPK phosphorylation, inhibited mTORC1 in the liver. **Conclusions**: These findings suggest that aerobic exercise alleviates oxidative stress and inflammation in NAFLD, with Sestrin2 activation playing a central role.

## 1. Introduction

Non-alcoholic fatty liver disease (NAFLD) is a prevalent chronic liver disorder, affecting approximately 30% of the global population, with its prevalence continuously increasing [1]. NAFLD spans a range of conditions, from benign hepatic steatosis to the more progressive non-alcoholic steatohepatitis (NASH), which is marked by hepatocellular damage, lobular inflammation, and fibrosis [2]. The pathogenesis of NAFLD is multifactorial, with oxidative stress and chronic low-grade inflammation identified as key drivers of disease onset and progression [3,4]. Lipid accumulation in NAFLD is mainly attributed to dysregulated triglyceride (TG) metabolism [5]. Excess of fatty acids in hepatocytes overwhelm mitochondrial oxidative capacity, disrupting the balance between oxidants and antioxidants, thus inducing oxidative stress. Simultaneously, lipid accumulation triggers inflammatory signaling pathways, leading to the upregulation of pro-inflammatory cytokines [6]. The combined effects of oxidative stress and inflammation exacerbate liver injury, ultimately contributing to the development of hepatic fibrosis and cirrhosis. Given the central roles of oxidative stress and inflammation in NAFLD progression, endogenous regulators that modulate these processes may offer promising therapeutic targets.

Sestrins are a highly conserved family of stress-inducible proteins found in vertebrates and are known to exert protective effects by regulating oxidative stress, inflammation, autophagy, and endoplasmic reticulum (ER) stress [7]. Accumulating evidence supports the protective role of Sestrin2 across diverse pathological conditions. For instance, Sestrin2 activation reduces hepatic susceptibility to oxidative damage by regulating nuclear factor-erythroid 2-related factor 2 (Nrf2) [8]. Nrf2, in turn, directly regulates heme oxygenase-1 (HO-1), and the Nrf2/HO-1 axis exerts protective effects against oxidative stress-induced damage [9]. Moreover, Sestrins contribute to the prevention of NAFLD-related pathologies by modulating key signaling pathways, including AMPK and mTORC1 [10]. Activation of mTORC1 promotes the M1-like pro-inflammatory polarization of macrophages [11,12]. Conversely, mTORC1 inhibition facilitates M2-like macrophage polarization, which plays a key role in mitigating inflammation [13]. In macrophages, Sestrin2 significantly reduces lipopolysaccharide (LPS)-induced nitric oxide (NO) release, inducible nitric oxide synthase (iNOS) expression, and production of pro-inflammatory cytokines [14]. Additionally, Sestrin2 can suppress mTORC1-mediated inflammatory responses in M1 macrophages while promoting M2-like macrophage polarization [15]. Exercise has been recognized as an intervention for preventing and managing NAFLD [16]. Regular aerobic exercise effectively mitigates oxidative stress and chronic inflammation [17,18], and emerging evidence indicates that exercise can upregulate Sestrin2 expression [19].

In this study, we investigated the effect of aerobic exercise on NAFLD induced by a high-fat diet in *ApoE^-/-^* mice. We hypothesized that aerobic exercise would promote the increase in Sestrin2 expression, thereby alleviating oxidative stress and inflammatory responses. This would provide a new explanation for the improvement of metabolic-related diseases by exercise and highlight Sestrin2 as a promising target for drug intervention.

## 2. Materials and Methods

### 2.1. Experimental Animals

In this study, twelve 6-week-old male specific pathogen-free (SPF) C57BL/6J mice, serving as wild-type controls, were randomly divided into a sedentary control group (C, n = 6) and an aerobic exercise group (CE, n = 6). Another twelve age-matched male SPF *ApoE^-/-^* mice were employed to induce the NAFLD model and were randomly assigned to a sedentary *ApoE^-/-^* group (AS, n = 6) and an aerobic exercise *ApoE^-/-^* group (AE, n = 6). All mice were maintained under SPF conditions at the Animal Center of Zhejiang Normal University. A high-fat diet (HFD; #D12108C, Research Diets, New Brunswick, NJ, USA) containing 20% protein, 40% carbohydrate, and 40% fat was provided to the AS and AE groups, whereas the C and CE groups received a standard chow diet. A 12-week treadmill running protocol was performed on the mice in the CE and AE groups. Body weight was recorded weekly, and prior to the first exercise session, the average body weight across all groups ranged from (22.7–23.3 g). The Animal Care and Use Committee of Zhejiang Normal University approved all animal procedures (Protocol Approval No. ZSDW2024025).

### 2.2. Exercise Protocol

The exercise protocol in this study was modified based on previous protocols [20]. On Day 1, mice in the CE and AE groups ran at 10 m/min for 10 min to acclimatize to the treadmill. From Day 2 onwards, the running speed and duration were progressively increased by 1 m/min and 10 min per day, respectively, until reaching the target intensity of 15 m/min for 60 min per session. This intensity corresponds to approximately 60% of the maximal oxygen consumption (VO_2_max) in mice, based on a previous study using a similar running protocol [21]. Each 60 min treadmill session, performed at a 5° incline, included a 2 min rest interval after every 15 min of exercise. This protocol was conducted daily starting at 5:00 PM, 5 days per week, for 12 weeks.

### 2.3. Serum Biochemical Measurements

Blood was collected via abdominal aorta puncture under deep anesthesia, after which liver tissues were rapidly harvested. Serum concentrations of triglyceride (TG), total cholesterol (TC), low-density lipoprotein cholesterol (LDL-C), high-density lipoprotein cholesterol (HDL-C), interleukin-10 (IL-10), tumor necrosis factor-alpha (TNF-α), malondialdehyde (MDA), superoxide dismutase (SOD), alanine aminotransferase (ALT), and aspartate aminotransferase (AST) were determined using commercially available ELISA kits (Nanjing Jiancheng Bioengineering Institute, Nanjing, China), strictly following the protocols provided by the manufacturer. A Multiskan SkyHigh microplate reader (A51119700DPC, Thermo Fisher Scientific, Waltham, MA, USA) was employed to read the absorbance.

### 2.4. Histological Analysis

Hematoxylin–Eosin (H&E) Staining: Following a standard protocol, paraffin-embedded liver sections underwent deparaffinization in xylene, rehydration through a graded ethanol series, and staining with hematoxylin and eosin (H&E). After being dehydrated and cleared, the sections were mounted with neutral resin and observed under a light microscope (LEICA DM3000 LED, Wetzlar, Germany).

Oil Red O Staining: Liver tissues were sectioned at 10 μm thickness using a cryostat and assessed for lipid deposition via an improved Oil Red O (ORO) staining kit (G1261, Solarbio, Beijing, China). The stained sections were mounted and visualized under a light microscope (LEICA DM3000 LED).

### 2.5. Immunofluorescence Staining

A sodium citrate-based solution was employed for antigen retrieval on liver sections. Blocking was then carried out with 5% normal goat serum for 30 min at ambient temperature. After rinsing with PBS, the sections were subjected to overnight incubation with primary antibodies targeting iNOS (Proteintech, Rosemont, IL, USA, #18985-1-AP) and CD206 (Proteintech, #18704-1-AP) at 4 °C. The following day, a 1 h incubation with Alexa Fluor 488-conjugated secondary antibody (Abcam, Waltham, MA, USA, #ab150077) was conducted at room temperature. Nuclear counterstaining was achieved using DAPI (Beyotime Biotechnology, Shanghai, China), and fluorescence visualization was performed on a Leica fluorescence microscope (LEICA DM3000 LED).

### 2.6. Quantitative Real-Time PCR (qPCR)

Total RNA was isolated from liver tissue with TRIzol reagent (15596026CN, Thermo Fisher Scientific). After conventional cDNA synthesis, real-time PCR was performed using the primer sequences shown in Table 1, and relative mRNA expression was quantified via the 2^−ΔΔCt^ method.

### 2.7. Western Blot

Equal amounts of protein were resolved by SDS-PAGE and transferred onto PVDF membranes. The membranes were then incubated overnight at 4 °C with primary antibodies against the following targets: Sestrin2, HO-1, Keap1, SOD1, Catalase, IL-1β, and GAPDH (Proteintech, Rosemont, IL, USA; #10795-1-AP, #10701-1-AP, #10503-2-AP, #10269-1-AP, #21260-1-AP, #16806-1-AP, #60004-1-Ig, respectively); p-AMPKα (Thr172), AMPKα, mTOR, and Raptor (Cell Signaling Technology, Danvers, MA, USA; #2535, #2532, #2983, #2280); and Nrf2, TNF-α, and β-actin (ABclonal, Wuhan, China; #A21176, #A24214, #AC026). Following washing, HRP-conjugated secondary antibodies were applied for 2 h at room temperature. Protein bands were visualized using ECL reagent (Thermo Fisher Scientific) and quantified by densitometry with ImageJ software (Ver 1.51-java 8, Bethesda, MD, USA).

### 2.8. Statistical Analysis

Data were analyzed by GraphPad Prism 9.5 (Version 9.5, San Diego, CA, USA). Prior to statistical analysis, normality of data distribution was assessed using the Shapiro–Wilk test, and homogeneity of variances was evaluated using Levene’s test. Parametric tests (two-way ANOVA with Tukey’s post hoc test) were used when the data met the assumptions of normality and homogeneity of variance; otherwise, non-parametric alternatives were employed. Results are expressed as mean ± SEM. Statistical significance was set at *p* < 0.05.

## 3. Results

### 3.1. Aerobic Exercise Attenuates Hepatic Injury Markers and Serum Lipid Levels in ApoE^-/-^ Mice

Table 2 presents the serum biochemical data, demonstrating that the AS group had markedly higher TC, TG, LDL-C, AST, and ALT levels than the C group (*p* < 0.05). In contrast, the AE group showed a marked reduction in TC, TG, LDL-C, AST, and ALT levels (*p* < 0.05) and a significant increase in HDL-C (*p* < 0.05) compared to the AS group.

### 3.2. Aerobic Exercise Ameliorates Hepatic Lipid Deposition and Steatosis in ApoE^-/-^ Mice

**Figure 1:**
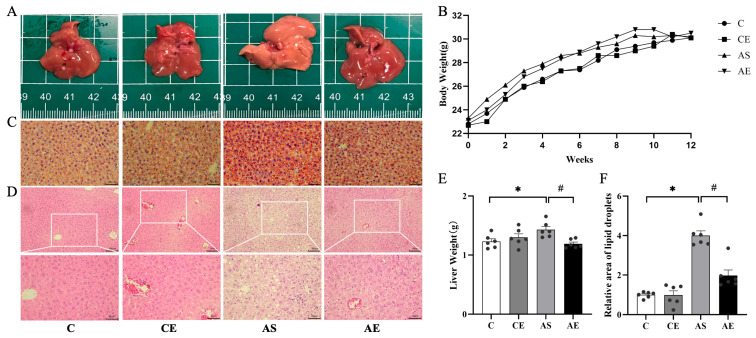
Aerobic exercise improves lipid deposition and fatty lesions in the liver of *ApoE^-/-^* mice. (**A**) Image of the liver tissue appearance. (**B**) Weight changes in mice in each group over 12 weeks. (**C**) Representative image of liver tissue ORO staining (400×). (**D**) Representative liver sections stained with H&E in the liver (200× and 400×). (**E**) Liver weight of each group of mice. (**F**) Quantification of Oil Red O. All data are presented as (mean ± SEM), and compared with group C: * *p* < 0.05; compared with AS group: ^#^ *p* < 0.05.

Body weight was comparable among all groups (Figure 1B). Livers from the C and CE groups were dark red with smooth surfaces. In contrast, the AS group livers were pale yellow (Figure 1A) and exhibited a significant increase in liver weight (*p* < 0.05; Figure 1E). After aerobic exercise, the AE group showed a restored dark red liver appearance (Figure 1A) and a significant reduction in liver weight (*p* < 0.05; Figure 1E). ORO staining (Figure 1C) revealed substantial lipid accumulation in the livers of the AS group compared to the C group (*p* < 0.05; Figure 1F), whereas aerobic exercise significantly reduced lipid deposition in the AE group relative to the AS group (*p* < 0.05; Figure 1F). H&E staining (Figure 1D) showed normal hepatic architecture in the C and CE groups, with intact lobular structure and orderly hepatocyte arrangement, along with only occasional cytoplasmic vacuoles. In contrast, the AS group exhibited hepatocyte swelling, disorganized cord arrangement, narrowed sinusoids, prominent ballooning degeneration, and abundant fatty vacuoles. The AE group demonstrated alleviation of sinusoidal narrowing and hepatocyte disarray, as well as a notable reduction in ballooning degeneration and lipid vacuoles.

### 3.3. Aerobic Exercise Attenuates Hepatic Oxidative Stress in ApoE^-/-^ Mice

**Figure 2:**
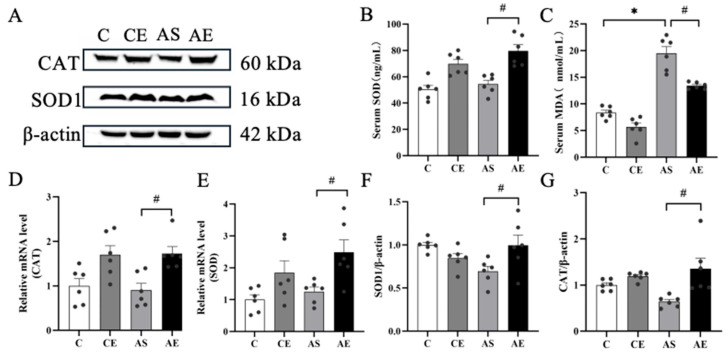
Aerobic exercise alleviates oxidative stress in the liver of *ApoE^-/-^* mice. (**A**) Western blot images of CAT and SOD1 proteins in liver tissues. (**B**,**C**) Contents of SOD and MDA in serum. (**D**,**E**) Expression levels of CAT and SOD-related mRNAs in liver tissues. (**F**,**G**) Expression levels of CAT and SOD1 proteins. All data are presented as (mean ± SEM), and compared with group C: * *p* < 0.05; compared with group AS: ^#^ *p* < 0.05.

Serum levels of MDA were significantly elevated in the AS group compared to the C group (*p* < 0.05; Figure 2C). After 12 weeks of aerobic exercise, serum MDA levels were significantly reduced (*p* < 0.05; Figure 2C), while SOD activity was significantly increased (*p* < 0.05; Figure 2B). Further analysis of mRNA and protein levels of key antioxidant enzymes revealed that, compared to the AS group, the AE group showed significantly higher mRNA expression of SOD and CAT (*p* < 0.05; Figure 2D,E), along with a concurrent increase in their respective protein levels (*p* < 0.05; Figure 2F,G).

### 3.4. Aerobic Exercise Alleviates Hepatic Inflammation in ApoE^-/-^ Mice

**Figure 3:**
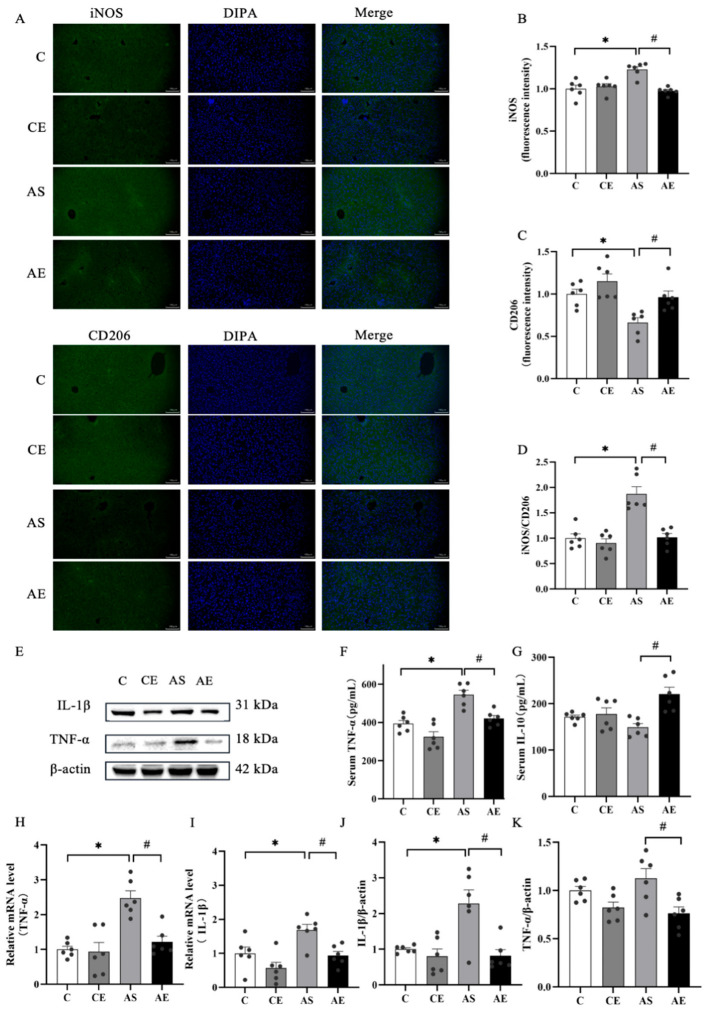
Aerobic exercise alleviates liver inflammation in *ApoE^-/-^* mice. (**A**) Representative fluorescence images of iNOS and CD206 in liver tissue. (**B**) Relative fluorescence intensity expression of iNOS. (**C**) Relative fluorescence intensity expression of CD206. (**D**) Relative ratio of iNOS/CD206. (**E**) Representative images of IL-1β and TNF-α protein blotting. (**F**,**G**) Levels of IL-10 and TNF-α in serum. (**H**,**I**) mRNA levels of IL-1β and TNF-α. (**J**,**K**) Protein expression levels of IL-1β and TNF-α. All data are presented as (mean ± SEM), and compared with group C: * *p* < 0.05; compared with group AS: ^#^ *p* < 0.05.

No significant differences in iNOS and CD206 fluorescence intensity were observed between the C and CE groups. However, the AS group showed a marked upregulation of iNOS expression and the iNOS/CD206 ratio, along with a downregulation of CD206 compared to the C group (*p* < 0.05; Figure 3B,D). Aerobic exercise significantly increased CD206 expression and decreased iNOS expression and the iNOS/CD206 ratio in the AE group (*p* < 0.05; Figure 3C,D).

Additionally, serum TNF-α levels were significantly higher in the AS group (*p* < 0.05; Figure 3F), whereas aerobic exercise notably reduced TNF-α levels and increased IL-10 levels (*p* < 0.05; Figure 3F,G). To further investigate the impact of aerobic exercise on inflammation, mRNA and protein levels of key inflammatory mediators were examined in liver tissue. Compared to the C group, the AS group showed significantly higher mRNA and protein levels of TNF-α and IL-1β (*p* < 0.05; Figure 3H–K). In contrast, aerobic exercise significantly reduced both mRNA and protein levels of TNF-α and IL-1β in the AE group (*p* < 0.05; Figure 3H–K).

### 3.5. Aerobic Exercise Upregulated the Expression of Regulation Oxidative Stress Related Proteins in ApoE^-/-^ Mice

**Figure 4:**
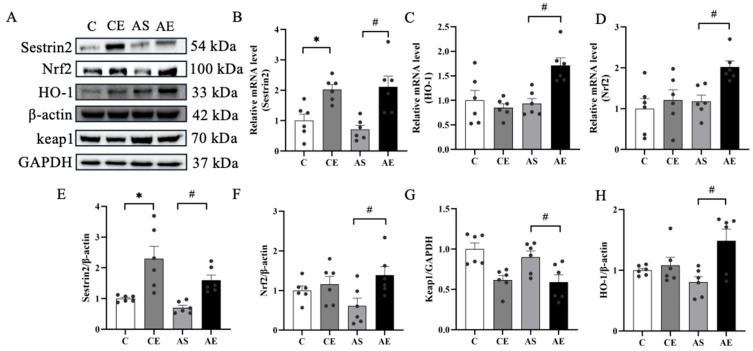
Aerobic exercise upregulated the expression of regulation oxidative stress related proteins in *ApoE^-/-^* Mice. (**A**) Representative image of protein blotting, including Sestrin2, Nrf2, HO-1, Keap1, and reference β-actin and GAPDH. (**B**–**D**) mRNA levels of related proteins, including Sestrin2, Nrf2, and HO-1. (**E**–**H**) Protein expression levels of Sestrin2, Nrf2, HO-1, and Keap1. All data are presented as (mean ± SEM), and compared with group C: * *p* < 0.05; compared with group AS: ^#^ *p* < 0.05.

To assess the effect of aerobic exercise on Sestrin2, its expression in liver tissue was analyzed via Western blot and quantitative RT-PCR. Aerobic exercise significantly increased both mRNA and protein levels of Sestrin2 in the CE and AE groups compared to their respective sedentary controls (*p* < 0.05; Figure 4B,E). The impact of exercise on proteins related to the Nrf2/HO-1 pathway was also evaluated. Compared to the AS group, the AE group exhibited elevated mRNA and protein levels of Nrf2 and HO-1 (*p* < 0.05; Figure 4C,D,F,H), accompanied by a marked reduction in Keap1 protein expression (*p* < 0.05; Figure 4G).

### 3.6. Aerobic Exercise Downregulated the Expression of Regulation Inflammation Related Proteins in ApoE^-/-^ Mice

**Figure 5:**
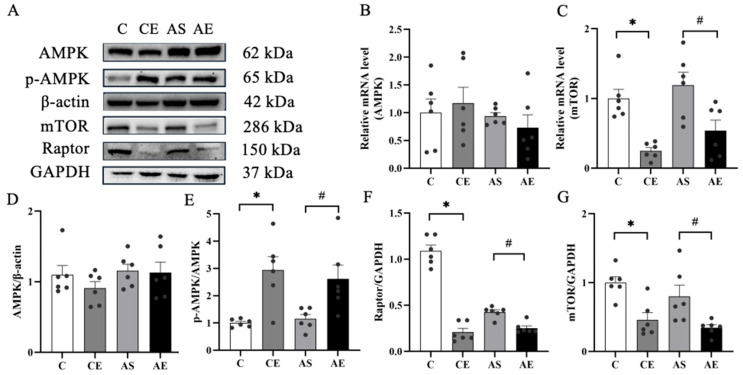
Aerobic exercise downregulated the expression of regulation inflammation related proteins in *ApoE*^-/-^ mice. (**A**) Representative image of protein blotting, including AMPK, mTOR, Raptor and reference β-actin and GAPDH. (**B**,**C**) mRNA levels of AMPK and mTOR. (**D**–**G**) Protein expression levels of AMPK, p-AMPK, mTOR, and Raptor. All data are presented as (mean ± SEM), and compared with group C, * *p* < 0.05; compared with group AS, ^#^ *p* < 0.05.

As a central regulatory hub, mTORC1 orchestrates inflammatory responses through its interaction with AMPK and downstream effectors. Our results showed that, compared to the C group, aerobic exercise significantly increased the p-AMPK/AMPK ratio in the CE group (*p* < 0.05; Figure 5E) and reduced protein levels of mTOR and Raptor (*p* < 0.05; Figure 5F,G). Similarly, compared to the AS group, the AE group exhibited a significantly higher p-AMPK/AMPK ratio (*p* < 0.05; Figure 5E) and markedly decreased protein expression of mTOR and Raptor (*p* < 0.05; Figure 5F,G). mTOR mRNA expression was also evaluated, revealing a significant reduction in the CE group compared to the C group (*p* < 0.05; Figure 5C) and a significant decrease in the AE group relative to the AS group (*p* < 0.05; Figure 5C).

## 4. Discussion

This study aimed to determine whether aerobic exercise alleviates NAFLD in *ApoE^-/-^* mice through Sestrin2 activation, a molecular mechanism with therapeutic relevance given current treatment limitations. Previous studies have demonstrated that *ApoE^-/-^* mice develop NAFLD after 7 weeks of HFD feeding [22,23]. In our study, serum cholesterol levels were significantly elevated in *ApoE^-/-^* mice, accompanied by marked hepatic lipid deposition and hepatocyte ballooning. Additionally, serum AST and ALT levels were notably increased, indicating the classic NAFLD phenotype (Table 2). 12 weeks of aerobic exercise significantly ameliorated these liver function abnormalities, which aligns with previous reports [24,25,26]. These findings support the conclusion that aerobic exercise alleviates NAFLD-associated liver injury, likely by improving lipid metabolism. We therefore recommend aerobic exercise as an effective intervention for NAFLD. As a chronic metabolic disorder, NAFLD is pathologically defined by excessive intrahepatic lipid accumulation and progressive hepatic dysfunction [27]. Our findings reveal that the wet weight of the liver was significantly elevated in *ApoE^-/-^* mice. However, following 12 weeks of aerobic exercise, liver weight decreased significantly (Figure 1). This reduction is likely attributable to the effect of aerobic exercise in reducing hepatic fat accumulation. Interestingly, no significant changes in body weight were observed in any group of mice after aerobic exercise. Similarly, Wu et al. reported that aerobic exercise did not alter body weight in ApoE-deficient mice [28]. We propose that this stability in overall body weight is likely due to exercise-induced changes in body composition.

NAFLD is primarily driven by excessive hepatic lipid accumulation, primarily TG [29]. When lipid overload exceeds the liver’s metabolic capacity, mitochondrial dysfunction ensues, leading to increased ROS production during fatty acid oxidation (FAO) and resulting in oxidative stress [30]. Oxidative stress is a well-established contributor to NAFLD pathogenesis [31], and maintaining precise redox homeostasis is essential for sustaining hepatic lipid metabolism. Aerobic exercise is widely recommended as a cornerstone non-pharmacological intervention for NAFLD [32]. In this study, 12 weeks of aerobic exercise significantly reduced serum TG concentrations in *ApoE^-/-^* mice. Additionally, this study assessed its impact on the hepatic antioxidant defense system, observing that aerobic exercise significantly upregulated both mRNA and protein expression of SOD1 and CAT in the liver, while concurrently lowering serum MDA levels, a key marker of lipid peroxidation (Figure 2). These results are consistent with previous findings showing that aerobic exercise reduces serum TG and TC in HFD-induced NAFLD models [24,33,34]. Mechanistically, studies have demonstrated that chronic exercise enhances mitochondrial β-oxidation efficiency [35], thereby reducing ectopic lipid deposition and associated oxidative injury [36]. In summary, 12 weeks of aerobic exercise significantly mitigates hepatic oxidative stress in *ApoE^-/-^* mice via enhanced antioxidant defenses and reduced lipid accumulation, positioning it as an effective non-pharmacological strategy for NAFLD management.

Sestrin2 is a stress-inducible, evolutionarily conserved protein with potent antioxidant properties [37]. Increasing evidence suggests that Sestrin2 activates the Nrf2 pathway by promoting Keap1 degradation [38,39], thereby reducing hepatic oxidative damage [40]. However, the molecular mechanisms through which aerobic exercise alleviates NAFLD remain incompletely understood. A previous study has shown that chronic physical training increases Sestrin2 protein levels in skeletal muscle [41]. Similarly, our data demonstrate that 12 weeks of aerobic exercise significantly upregulates both the protein and mRNA expression of Sestrin2 in the liver of *ApoE^-/-^* mice. Further, this study investigated the hepatic Nrf2/HO-1 antioxidant signaling axis and found that aerobic exercise significantly enhanced the expression of proteins associated with this pathway in *ApoE^-/-^* mice. Consistent with previous report that long-term aerobic exercise activates Nrf2 and induces antioxidant enzymes like HO-1 [42], our results confirm that this pathway plays a pivotal role in strengthening hepatic antioxidant defenses.

Prolonged oxidative stress not only leads to hepatocyte injury but also triggers inflammatory responses [43]. The mTORC1 signaling complex regulates inflammatory responses through various mechanisms, notably by promoting pro-inflammatory M1 macrophage polarization [44]. In this study, significant M1 macrophage polarization was observed in *ApoE^-/-^* mice. According to a previous study, M1 macrophage activation leads to the release of pro-inflammatory cytokines, initiating inflammation [45]. A preclinical study also confirms that liver-resident macrophages in patients with NAFLD predominantly exhibit an M1-like phenotype [46]. In contrast, activation of M2 macrophages alleviates hepatic steatosis and inflammation [47]. Thus, rebalancing macrophage polarization represents a promising therapeutic strategy for NAFLD. Notably, aerobic exercise restores M1/M2 equilibrium in macrophages and suppresses associated inflammatory pathways [48]. Our findings support this, demonstrating that aerobic exercise promoted macrophage polarization toward the M2 phenotype in *ApoE^-/-^* mice.

To investigate whether Sestrin2 induction contributes to the anti-inflammatory effects of aerobic exercise in NAFLD, key inflammatory mediators in liver tissue and serum were quantified. The results showed significantly elevated levels of inflammatory factors such as TNF-α and IL-1β in the livers of *ApoE^-/-^* mice. However, after 12 weeks of aerobic exercise, serum IL-10 levels increased, while pro-inflammatory factors, including TNF-α and IL-1β, were reduced in liver tissue. Mechanistically, mTORC1 signaling plays a pivotal role in regulating macrophage polarization and function [49]. It is well established that Sestrin2 activates AMPK while simultaneously inhibiting mTORC1 signaling [50]. These findings align with existing mechanistic frameworks, demonstrating that 12 weeks of aerobic exercise significantly attenuated hepatic inflammation in *ApoE^-/-^* mice (Figure 3). This anti-inflammatory effect likely involves, at least in part, the inhibition of mTORC1 signaling following Sestrin2 activation.

## 5. Limitations of the Study

This study has several limitations. While a positive correlation between Sestrin2 upregulation and activation of the Nrf2/HO-1 and AMPK/mTORC1 pathways was observed, the functional necessity of Sestrin2 was not directly verified through gene knockout or specific inhibition experiments. Future studies involving Sestrin2-specific knockout or inhibition are needed to confirm its indispensable role and further elucidate its downstream effectors.

## 6. Conclusions

In conclusion, our results demonstrate that 12 weeks of aerobic exercise significantly alleviates NAFLD-associated hepatic dysfunction, lipid accumulation, oxidative stress, and inflammation. These protective effects are closely associated with the activation of Sestrin2-mediated antioxidant and anti-inflammatory signaling pathways. Therefore, we propose that Sestrin2 plays a key regulatory role in the amelioration of NAFLD by aerobic exercise, providing experimental evidence for its potential as a therapeutic target.

## 7. Suggestions and Practical Applications

Based on our findings, we recommend aerobic exercise as an effective non-pharmacological intervention for NAFLD. Additionally, the Sestrin2-mediated antioxidant and anti-inflammatory pathways identified in this study may represent promising targets for future drug development. Future research is warranted to investigate the potential synergy between such agents and exercise interventions.

## Figures and Tables

**Table 1:** Primer sequences.

Gene	Primer Forward	Primer Reverse
GAPDH	GCCTCCTCCAATTCAACCCT	CTCGTGGTTCACACCCATCA
Sestrin2	ACTGCGTCTTTGGCATCAGA	CCCTTGGCCTTTCCGAATCT
Nrf2	TCTCCTAGTTCTCCGCTGCT	GGTTACAACGTGGGGATGGT
HO-1	GTCAGGTGTCCAGAGAAGGC	CATCACCTGCAGCTCCTCAA
AMPK	GTACCAGGTCATCAGTACACCA	GTGGACCACCATATGCCTGT
mTOR	ACAGATCCTGGTCTTTGAGATCC	AGCCTTCAGGATAGGCTCCA
SOD	GGAACCATCCACTTCGAGCA	CCCATGCTGGCCTTCAGTTA
CAT	AGGCTCAGCTGACACAGTTC	ATGGAGAGACTCGGGACGAA
TNF-α	ACCCTCACACTCACAAACCA	ACCCTGAGCCATAATCCCCT
IL-β	TGCCACCTTTTGACAGTGATG	TTCTTGTGACCCTGAGCGAC

**Table 2:** Levels of serum TC, TG, LDL-C, HDL-C, ALT, and AST (mean ± SEM).

Groups	C	CE	AS	AE
TC (mmol/L)	2.25 ± 0.22	2.42 ± 0.24	20.01 ± 0.97 *	14.46 ± 0.63 **^#^**
TG (mmol/L)	0.57 ± 0.08	0.59 ± 0.05	1.09 ± 0.06 *	0.77 ± 0.05 **^#^**
LDL-C (mmol/L)	2.60 ± 0.33	2.68 ± 0.11	14.44 ± 0.89 *	11.22 ± 0.76 **^#^**
HDL-C (mmol/L)	2.29 ± 0.18	3.23 ± 0.29 *	1.68 ± 0.07	3.23 ± 0.19 **^#^**
ALT (U/L)	12.06 ± 1.84	11.50 ± 1.10	41.42 ± 5.21 *	17.54 ± 1.34 **^#^**
AST (U/L)	7.05 ± 1.05	7.25 ± 0.72	17.72 ± 2.42 *	11.58 ± 0.51 **^#^**

Compared with Group C, * *p* < 0.05; compared with the AS group, ^#^ *p* < 0.05.

## Data Availability

The findings of this study are supported by data that are accessible upon request from the corresponding author.

## Abbreviations

The following abbreviations are used in this manuscript:

TC	Total cholesterol
TG	Triglycerides
LDL-C	Low-density lipoprotein cholesterol
HDL-C	High-density lipoprotein cholesterol
AST	Aspartate aminotransferase
ALT	Alanine aminotransferase
NAFLD	Non-alcoholic fatty liver disease
NASH	Non-alcoholic steatohepatitis
MDA	Malondialdehyde
SOD	Superoxide dismutase
IL-10	Interleukin-10
IL-1β	Interleukin-1beta
TNF-α	Tumor necrosis factor-alpha
Keap1	Kelch-like ECH-associated protein 1
Nrf2	Nuclear factor-erythroid 2-related factor 2
HO-1	Heme oxygenase-1
AMPK	Adenosine 5′-monophosphate (AMP)-activated protein kinase
p-AMPK	phosphorylated AMP-activated protein kinase
mTOR	mechanistic Target of Rapamycin
mTORC1	mechanistic target of rapamycin complex 1
HFD	High-fat diet
FAO	Fatty acid oxidation
